# Seasonal Patterns of Dominant Microbes Involved in Central Nutrient Cycles in the Subsurface

**DOI:** 10.3390/microorganisms8111694

**Published:** 2020-10-30

**Authors:** Patrick Lohmann, Simon Benk, Gerd Gleixner, Karin Potthast, Beate Michalzik, Nico Jehmlich, Martin von Bergen

**Affiliations:** 1 Department of Molecular Systems Biology, Helmholtz-Centre for Environmental Research GmbH—UFZ, 04318 Leipzig, Germany; patrick.lohmann@ufz.de (P.L.); nico.jehmlich@ufz.de (N.J.); 2 Department of Molecular Biogeochemistry, Max-Planck-Institute for Biogeochemistry, 07745 Jena, Germany; sbenk@bgc-jena.mpg.de (S.B.); gerd.gleixner@bgc-jena.mpg.de (G.G.); 3 Department of Soil Science, Friedrich Schiller University, 07743 Jena, Germany; karin.potthast@uni-jena.de (K.P.); beate.michalzik@uni-jena.de (B.M.); 4 German Center for Integrative Biodiversity Research (iDiv) Halle-Jena-Leipzig, 04103 Leipzig, Germany; 5 Institute of Biochemistry, Faculty of Biosciences, Pharmacy and Psychology, University of Leipzig, 04103 Leipzig, Germany

**Keywords:** metaproteomics, microbial communities, subsurface, nutrient cycles, critical zone

## Abstract

Microbial communities play a key role for central biogeochemical cycles in the subsurface. Little is known about whether short-term seasonal drought and rewetting events influence the dominant microbes involved in C- and N-cycles. Here, we applied metaproteomics at different subsurface sites in winter, summer and autumn from surface litter layer, seepage water at increasing subsoil depths and remote located groundwater from two wells within the Hainich Critical Zone Exploratory, Germany. We observed changes in the dominance of microbial families at subsurface sampling sites with increasing distances, i.e., *Microcoleaceae* dominated in topsoil seepage, while *Candidatus Brocadiaceae* dominated at deeper and more distant groundwater wells. Nitrifying bacteria showed a shift in dominance from drought to rewetting events from summer by *Nitrosomandaceae* to autumn by *Candidatus Brocadiaceae*. We further observed that the reductive pentose phosphate pathway was a prominent CO_2_-fixation strategy, dominated by *Woeseiaceae* in wet early winter, which decreased under drought conditions and changed to a dominance of *Sphingobacteriaceae* under rewetting conditions. This study shows that increasing subsurface sites and rewetting event after drought alter the dominances of key subsurface microbes. This helps to predict the consequences of annual seasonal dynamics on the nutrient cycling microbes that contribute to ecosystem functioning.

## 1. Introduction

The earth’s Critical Zone (CZ) evolved as an emerging research area where the fundamental physical, chemical and biological processes take place [1,2]. The CZ ranges from the top of vegetation through the subsurface saturated and unsaturated zone down to aquifer systems [3]. The subsurface harbors more than half of the global existing microorganisms [4], which are physically and chemically associated to form complex microbial communities [5] that are capable of colonizing subsoil environments [6] to control key ecological processes [7].

Drought periods occur more frequently as a consequence of climate change and have been studied with regard to changes in subsurface microbial communities [8,9,10]. It has been shown that long-term droughts significantly affect microbial activity, biomass, and the composition of microbial communities in terrestrial ecosystems [11,12]. This has led to the physiological mechanism of community-level adaptations to long drought periods and had consequences on the stability and fertility of ecosystems [9,13]. It is expected that subsequent precipitation events after droughts will also increase in the future, which can lead to climate-induced shifts in the structure and composition of subsurface communities [14,15]. Changing precipitation patterns through drying and rewetting events select for drought-tolerant microbial taxa and, therefore, may result in a community that reacts differently to subsequent moisture stress [14]. Annual drought conditions in summer through decreases in precipitation and increases in water evaporation limit the topsoil seepage water that transports microbes and surface-derived nutrients through the subsurface [10]. It has been shown that variation of seasonal precipitation events changes the soil profile through dynamic carbon (C) and nitrogen (N) inputs, which affects microbial processes [16]. Seepage water determines the hydraulic head and recharges the nutrient content of deeper located groundwater [17,18]. However, it is yet not clear whether short-term annual fluctuations of drought and rewetting events influence the functionality and composition of seepage and groundwater living microbial communities involved in central biogeochemical processes [19].

Microbes, which are responsible for biogeochemical cycles, convert nitrogen and carbon for energy production. Changes in nutrient availability through dynamic precipitation regimes can alter these processes, leading to rapid adaptation of such dominant microbes [20,21,22]. Nutrient cycles are crucial for the biogeochemistry on earth since C and N are essential for the production of proteins, amino and nucleic acids [23]. They play an important role in determining the health status of an ecosystem, which can be traced through ecosystem services directly linked to microbial activities for the maintenance of human livelihoods [24]. Overall, microbes transform molecular N from the atmosphere into ammonium (NH_4_^+^) to make inaccessible nitrogen bioavailable to plants and other organisms [25,26]. Subsequent anaerobic ammonium oxidation (anammox) or denitrification release nitrogen back to the atmosphere, thus controlling nitrate levels in groundwater [27,28,29]. In addition, microbes control the fluxes of climate-relevant gases for autotrophic CO_2_-fixation. They assimilate atmospheric CO_2_ into cellular carbon to generate a potential carbon source [30,31]. It has been suggested that up to 17% of microbial communities in groundwater are involved in CO_2_-fixation [32], where carbon induced autotrophy is an important strategy for energy production in addition to nitrogen conversion in subsurface environments. In order to assess the taxonomic distribution; biochemical functions and systems-level microbial interactions of communities, a variety of high-throughput techniques have been evolved e.g., metaproteomics to analyze the entirety of proteins from microbial communities [25,33]. Although the gene content of microbes can provide structural information about a community, the measurement of proteins is arguably more reliable for characterizing their functionality [34]. 

Here, we applied metaproteomics to characterize the taxonomical and functional structure of microbial communities under drought and rewetting conditions from different subsurface sites of the Hainich Critical Zone Exploratory (CZE). We focused on the surface litter layer (0 cm) and three different seepage water sampling depths (4 cm, 16 cm and 30 cm) of two beech-dominated forest plots. Moreover, we sampled two groundwater wells H42 (12.7 m) and H52 (65 m) of a horizontal aquifer transect which is located downhill at an approximately 3.4 km distance to the seepage water collecting lysimeter. We hypothesized that (i) increasing subsurface distances show compositional and functional changes in the present microbial communities and that (ii) seasonal transition of drought to rewetting periods can alter the dominance of microbes involved in central processes of the nitrogen cycle and CO_2_-fixation.

## 2. Materials and Methods

### 2.1. Study Site and Sampling

**Figure 1:**
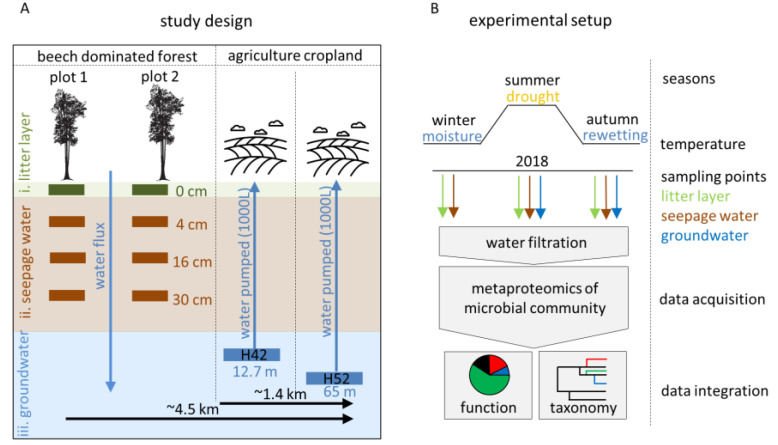
(**A**) Schematic overview of the Hainich Critical Zone Exploratory (CZE) with three sampling sites (i. litter layer, ii. seepage water, iii. groundwater) and depths (0–30 cm, 12.7 m and 65 m). The litter layer and seepage water was sampled at two plots with two replicates (*n* = 2). The filtered groundwater per well was used for two replicates (*n* = 2). (**B**) Experimental setup of the sampling strategy for a seasonal comparison of the microbial community.

Litter layer, seepage water and groundwater samples were collected from the Hainich CZE located in western Thuringia, Germany. This study site was established by the Collaborative Research Centre (CRC) AquaDiva [2]. The geological, lithological and hydrological composition of this study site is documented elsewhere in more detail [2,35]. The top slope area encompasses managed forest sites dominated by European beech (*Fagus sylvatica*) mixed with ash (*Fraxinus excelsior*) and maple trees (*Acer pseudoplatanus*) [36]. The free-drained lysimeter was installed to sample the seepage water from two plots and two replicates from the litter layer and mineral soil at 4, 16 and 30 cm depths (details are described in [36]) and sampling parameters are provided (Supplement Table S1). The groundwater wells (H1 to H5) along a 5.4 km transect provide access to a shallow groundwater flow system in sloping thin-bedded limestone-mudstone with an average slope of 35 m/km, which allows for sampling of groundwater from 2 to 85 m depths [35,37]. The Hainich transect is partitioned into a Trochitenkalk formation (moTK) and Meissner formation (moM) [35], which reflects anoxic to sub-oxic conditions and dominance of mudstone with a pH above 7.2 and electric conductivity exceeding 500 µS/cm [38]. In this study, we focused on the groundwater wells H42 in 12.7 m depths and H52 in 65 m depths covered by cropland from different seasons, which are around 1.4 km distantly located from each other and in about 3.4 km distance from the installed lysimeter to sample seepage water [39] (Figure 1A,B). The constant groundwater flow was sampled during regular sampling campaigns within the coordinated monitoring program of the CRC AquaDiva and pumped up to 1000 L to the surface by a submersible pump described in [40]. The water was filtered through a pre-combusted glass fiber filter with a diameter of 293 mm and 0.3 µm pores on a stainless filter holder to collect bacterial cells with a flow of about 20 L/m. The filters were stored on dry ice for further sample preparation steps in the laboratory. 

### 2.2. Bacterial Cell Lysis and Protein Extraction

Cells were resuspended in 1–5 mL Lysis-buffer (0.29% NaCl, 0.01 M Tris-HCl, 5 mM EDTA, 0.4% SDS, pH 6.8) with 1 µL PMSF solution. The suspended cells were further lysed by bead-beating with 3 cycles of FastPrep (MP Biomedicals, Santa Ana, CA, USA) for 1 min. The lysate was then heated and mixed for 15 min at 60 °C in a Thermomixer (Eppendorf, Hamburg, Germany). The cell debris was removed by centrifugation at 10,000× *g* for 10 min at 4 °C. The proteins were precipitated in 5 volumes of pre-cold acetone with an overnight incubation at −20 °C. The precipitated proteins were centrifuged at 15,000× *g* for 10 min at 4 °C. The pellet was evaporated using a SpeedVac (Eppendorf, Hamburg, Germany) for 5 min. The dry protein pellet was stored at −20 °C. 

### 2.3. SDS-PAGE, Proteolytic Digestion, and Peptide Extraction

For sodium dodecyl sulfate polyacrylamide gel electrophoresis (SDS-PAGE), the protein pellet was resuspended with 20 µL SDS loading buffer and incubated for 5 min in a Thermomixer at 95 °C and 1400 rpm. After SDS-PAGE and staining with colloidal Coomassie brilliant blue (Merck, Darmstadt, Germany) overnight, the colored gel bands containing all proteins were cut out and were sliced into smaller gel pieces. Then, the gel bands were destained by two rinses with H_2_O for 30 min at room temperature. Proteins in each band were modified with 10 mM Dithioerythritol (DTT) and 100 mM 2-iodacetamide (IAA) and incubated for 30 min at room temperature. We applied 20 µg alkylated proteins which were proteolytically digested using 0.5 µg trypsin (Sigma-Aldrich, St. Louis, MO, USA) at 37 °C, overnight. Digestion was stopped by adding 10 mM ammonium bicarbonate in 0.1% formic acid (FA). After peptide extraction using extraction buffer (50% acetonitrile and 5% formic acid), the samples were evaporated using a SpeedVac for 2 h and stored at −20 °C. The extracted peptides were desalted using ZipTip filter (Thermo Fischer Scientific, Waltham, MA, USA) following the manufacturer’s instructions. Peptides were dissolved in 0.1% FA and injected into the liquid chromatography–mass spectrometer.

### 2.4. Liquid Chromatography–Tandem Mass Spectrometry (LC-MS/MS)

Samples were analyzed using liquid chromatography (HPLC, Ultimate 3000 RSLCnano, Dionex/Thermo Fisher Scientific, Idstein, Germany) coupled via a TriVersa NanoMate (Advion, Ltd., Harlow, UK) source in LC chip coupling mode with a Q Exactive HF mass spectrometer (Thermo Fisher Scientific, Waltham, MA, USA). An amount of 5 µg were first loaded for 5 min on the precolumn (µ-pre-column, Acclaim PepMap C18, 2 cm, Thermo Scientific) at 4% mobile phase B (80% acetonitrile in water with 0.08% formic acid) and 96% mobile phase A (water with 0.1% formic acid) at a flow rate of 300 nL/min and 35 °C. Then, the peptides were eluted from the analytical column (Acclaim PepMap C18 LC column, 25 cm, Thermo Scientific) over a 180 min linear gradient of mobile phase B (4%–50%). An Orbitrap analyzer was used for MS and MS/MS scans with higher energy collision dissociation (HCD) fragmentation. MS scans were measured at a resolution of 120,000 in the scan range of 400–1600 *m/z*. Most intense peaks (charge state 2–7) were isolated for MS/MS scans by a quadrupole with an isolation window of 2 Da and were measured with a resolution of 15,000. The dynamic exclusion was set to 30 s with a +/−10 ppm tolerance. The automatic gain control target was set to 5 × 10^4^ with an injection time of 150 ms.

### 2.5. Data Analysis

The acquired raw data were searched against a site specific database by the search engine Sequest HT using proteome discoverer (v.2.2., Thermo Fischer Scientific, Waltham, MA, USA). This database was generated by metagenome sequencing of groundwater community and contains 1,254,597 protein coding sequences. The search settings were: Trypsin (full), precursor mass tolerance of 10 ppm and fragment mass tolerance of 0.02 Da. We considered only proteins with a false discovery rate (FDR) <1%. The identified proteins were filtered according to the following criteria: (i) at least 1 replicate shows an abundance value, (ii) proteins contained at least one unique peptide were considered, (iii) non-bacterial proteins were removed and (iv) proteins assigned to only one protein group ID were considered. The proteins were then grouped into protein groups according to the lowest common ancestor (lca) for the different taxonomic ranks. Protein groups containing proteins which were not assigned to the same taxon were annotated as heterogeneous. The number of protein groups with a unique taxon were counted (without heterogeneous). For functional annotations, the Kyoto Encyclopedia of Genes and Genomes (KEGG) database was used to assign a KEGG number representing a specific metabolic function to the identified protein groups [41]. We provided a Table S2 of identified proteins with annotated protein group ID, taxonomic and functional information as Supplementary Material. The panels were created by R v3.6.1 with the installed packages ggplot2, extrafont, export, reshape and readr.

### 2.6. Dissolved Organic Matter (DOM) Composition Measurement

DOM was extracted from duplicates of 10 L filtered groundwater (<0.3 µm) using a common solid-phase extraction protocol on PPL resin [42]. Together with the samples, procedural blanks of ultrapure water were extracted for each sampling campaign. The concentration of the extracts was adjusted to 20 mg/L in a 1:1 water and methanol solvent mixture. A total of 100 µL of DOM extract was directly injected into an Orbitrap Elite mass spectrometer (Thermo Fisher Scientific), operated with electrospray ionization (ESI) in negative ionization mode (ESI needle voltage 2.65 kV). A total of 100 scans of 175–1000 *m/z* were acquired per sample with detailed settings and sum formula assignment as previously described in [17,43]. Metabolic pathway information was gathered from the Kyoto Encyclopedia of Genes and Genomes (KEGG) using their application programming interface at https://www.kegg.jp/kegg/rest/ (access date: 2019-04-16) [41].

## 3. Results

### 3.1. A Global View on the Microbial Spatial-Temporal Distribution

**Figure 2:**
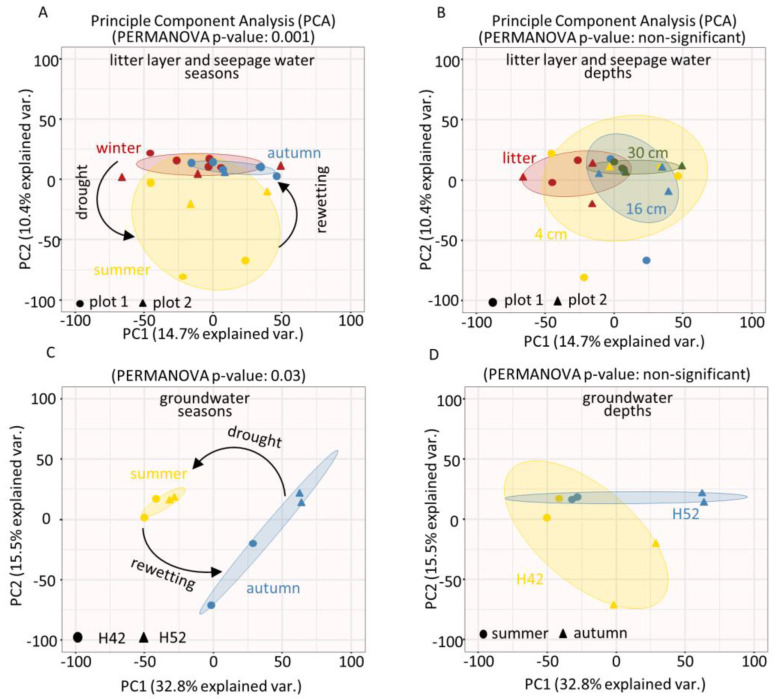
Principle component analysis (PCA) of abundance values of identified protein groups to compare (**A**) seasonal effects and (**B**) subsurface depths effects on litter layer and seepage water microbial community; and (**C**) seasonal effects and (**D**) subsurface depths effects on groundwater microbial community. *P*-values were calculated by PERMANOVA analysis.

To evaluate the separation of microbial communities in a spatial distribution by sampling depth and a temporal distribution by sampling time, we analyzed the composition of the community based on the relative abundances of the identified protein groups filtered according to criteria described in Section 2.5. In total, 6679 protein groups were identified and were used for the subsequent analysis. The Principal Component Analysis (PCA) showed a clear separation of the community concerning the drought summer season and also to the moist winter and rewetting autumn seasons in seepage water (PERMANOVA, *p*-value < 0.001) (Figure 2A).

This revealed a clear seasonal division of the microbial community from the near-surface seepage after the transition from drought to rewetting events. In contrast, the PCA showed an overall lower separation of the community with respect to the seepage water sampling depths (*p*-value: non-significant) (Figure 2B). For groundwater, the PCA also showed a separation of the community between drought and rewetting conditions (*p*-value: 0.03), as well as for the two wells H42 and H52 at 12.7 m and 65 m depth below the surface (*p*-value: non-significant) (Figure 2C,D). To better understand these differences in groundwater, a PCA analysis of dissolved organic matter (DOM) over multiple years showed that the DOM composition also changed seasonally, and was distinct between the two wells H42 and H52 (*p*-value < 0.001) (Figure S1A). Among the group of potentially microbial-derived compounds, especially nitrogen-containing molecules displayed clear seasonal differences for well H42 and slight differences for well H52 (Figure S1B). This supports our finding of a seasonally varying groundwater microbial community at the proteome level.

### 3.2. Taxonomic Characterization of the Subsurface Microbial Community

**Figure 3:**
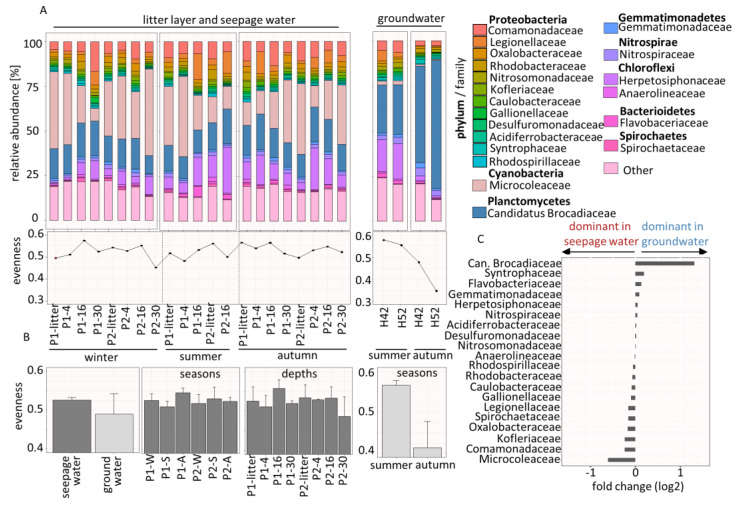
(**A**, **top**) stacked bar chart shows the mean relative abundance of families and (**A**, **bottom**) the evenness for litter layer, seepage water and groundwater based on identified protein groups, calculated by shannon-index/ln (family number); *n* = 2; P1 and 2 = plot1 and 2. (**B**) totalized evenness for seepage water and groundwater according to seasons and subsurface depths. (**C**) Fold change of log2 (intensity) to identify the families dominated in seepage water and groundwater.

The taxonomic profile of the community was characterized to assess possible compositional changes due to seasonal transition in the seepage and the remote groundwater wells H42 and H52. In total, we identified 266 families of which 106 families (39.8%) belong to the phylum *Proteobacteria*, the most dominant phylum in the subsurface (not shown). The top 20 abundant families were selected, representing the core community of which 11 families corresponded to the phylum *Proteobacteria* with a relative mean abundance of 1.7% and 1.2%; one family corresponded to the phylum *Cyanobacteria* with 21.8% and 1.3%; and one family corresponded to *Planctomycetes* with 12.8% and 38.4% for seepage water and groundwater, respectively (Figure 3A, top). In seepage water, we observed that the relative abundances of the top 20 families fluctuated with slight changes over the year from winter to autumn, which are not seasonally or depth-specific. In contrast, we found that *Candidatus Brocadiaceae* showed a strong increase in relative abundance from summer to autumn (22.5% to 54.2%) in the groundwater community. A subsequent evenness analysis was performed to reveal if the community consists of a few dominant microbes (by a low evenness value) or many equally frequent microbes (by a high evenness value) [44]. We observed that the evenness of the seepage community was not specifically influenced by seasonal changes or soil depth and showed fluctuations of an evenness value (ev) = 0.45 to 0.57. The evenness of the groundwater community decreased strongly from summer to autumn (ev = 0.58 to 0.35) (Figure 3A, bottom).

An overall comparison revealed a more even community in seepage water (ev = 0.53) compared to groundwater (ev = 0.49), while a comparison between the seasons and subsurface depths showed minor evenness changes (Figure 3B). Moreover, a fold change analysis revealed the families dominated either in seepage or in groundwater communities, including *Candidatus Brocadiaceae* (fold change (fc) = 1.14) as the most dominant family in groundwater, followed by *Syntrophaceae* (fc = 0.19) and *Flavobacteriaceae* (fc = 0.18). In contrast, seepage water was dominated by *Microcoleaceae* (fc = −0.65), followed by the *Comamonadaceae* (fc = −0.26) and the *Kofleriaceae* (fc = −0.21), which were rarely found in the groundwater (Figure 3C).

### 3.3. Functional Analysis of Pathways Relevant for Nutrient Cycles

**Figure 4:**
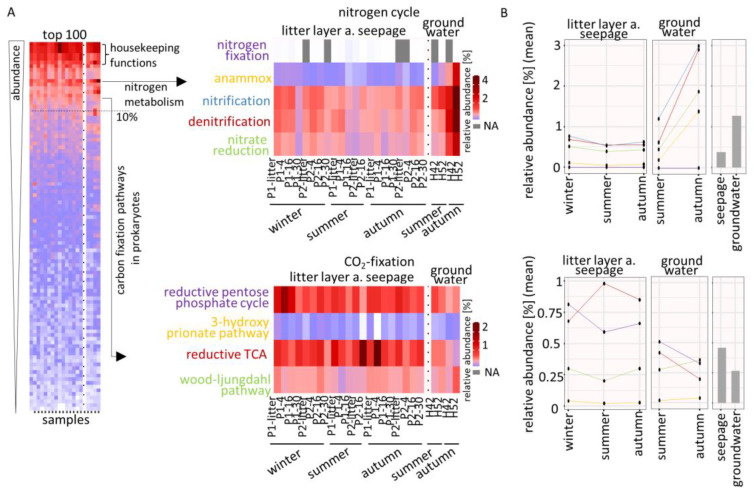
(**A**, **left**) Mean relative abundance of top 100 abundant pathways for litter layer, seepage water and groundwater based on identified protein groups. Dashed line represents the threshold for 10% of the highest abundant pathways. s = summer, a = autumn, P1 and 2 = plot1 and 2. (**A**, **right**) Relative abundance of the selected pathways clustering for the nitrogen cycle and CO_2_-fixation. (**B**) Mean relative abundance of the pathways belonging to nitrogen cycle and CO_2_-fixation for seepage water and groundwater according to the different seasons. Color code is represented by the pathways in A.

The 100 most abundant pathways were selected and displayed a differential abundance distribution, with most pathways showing low abundances. The most abundant pathways comprised general microbial life-sustaining metabolisms or housekeeping functions, e.g., translational metabolism and ribosomal pathways (Figure 4A). 

We observed that the nitrogen metabolism and carbon fixation pathways in prokaryotes belong to the 10% abundant pathways, which was a basic prerequisite for the following pathway analyses since these metabolisms include the pathways for nitrogen cycle und CO_2_-fixation. Furthermore, we found that the abundances of two CO_2_-fixation pathways increased seasonally, which means that the reductive pentose phosphate cycle was found as more abundant at the beginning of the year, while reductive TCA was more abundant later in the year (Figure 4B). In total, the pathways belonging to the nitrogen cycle were found as more abundant in groundwater (1.3%) compared to seepage (0.4%), while CO_2_-fixation pathways were found as more abundant in seepage (0.45%) compared to groundwater (0.27%). 

### 3.4. Seasonal Effects on Dominant Families Involved in Nitrogen Cycle and CO_2_-Fixation

**Figure 5:**
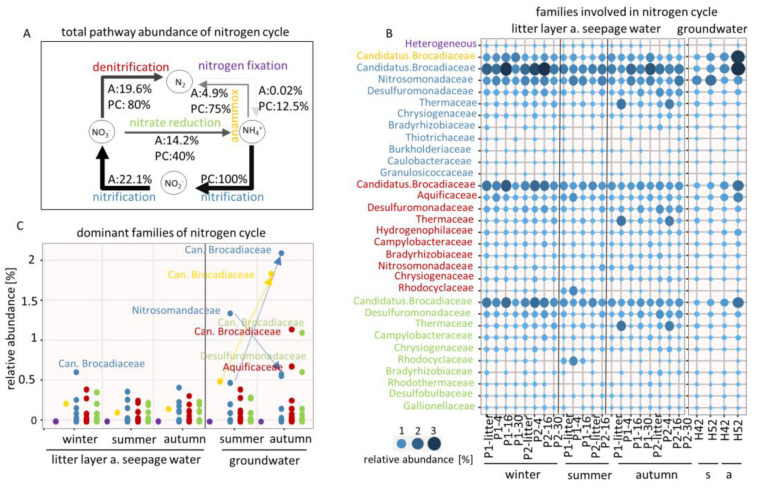
(**A**) Pathway abundance of the nitrogen cycle across the samples. A = abundance; PC = pathway coverage, calculated by identified number of proteins divided by total proteins per pathway. (**B**) Relative abundances of the families involved in nitrogen cycle. Colors represent the pathway affiliation; s = summer, a = autumn. P1,2 = plot1,2 (**C**) Relative abundances of the dominant families involved in nitrogen cycle in respect of the different seasons.

The nitrogen cycle pathways showed that nitrification (22.1%) was the most common pathway with a pathway coverage (PC) of 100%, closely followed by denitrification (19.6%, PC: 80%), nitrate reduction (14.2%, PC: 40%), anammox (4.9%, PC: 75%) and, as the least common pathway, nitrogen fixation (0.02%, PC: 12.5%) (Figure 5A). In general, *Candidatus Brocadiaceae* (3.1%) dominated all nitrogen cycle pathways except that of nitrogen fixation (Figure 5B). In particular, we found that the nitrification process was dominated by *Nitrosomandaceae* in summer under drought conditions with a decrease until autumn (1.4% to 0.6%), while *Candidatus Brocadiaceae* dominated under rewetting conditions in autumn with a decrease until summer (2.1% to 0.5%) (Figure 5C). For *Nitrosomandaceae* we identified the methane/ammonia monooxygenase with the subunits A, B and C (K10944; K10945 and K10946), which is responsible for the first step of nitrification. For *Candidatus Brocadiaceae*, we identified the hydroxylamine dehydrogenase (K10535) and the nitrate reductase/nitrite oxidoreductase, alpha-subunit (K00370), both of which are involved in both steps of nitrification. Denitrification and the anaerobic ammonia oxidation (anammox) process were rarely found under drought conditions while rewetting conditions in autumn revealed a dominance of *Candidatus Brocadiaceae* (1.2% and 1.8%, respectively). About the denitrification process, we identified the nitrate reductase/nitrite oxidoreductase, alpha-subunit (K00370) and nitrate reductase gamma subunit (K00374), while anammox process was identified by hydrazine synthase subunit A and B (K20934 and K20933). The family *Aquificaceae* (0.8%) was found to be the second most dominant microbe for denitrification in winter with the nitrite reductase (NO-forming)/hydroxylamine reductase (K15864).

**Figure 6:**
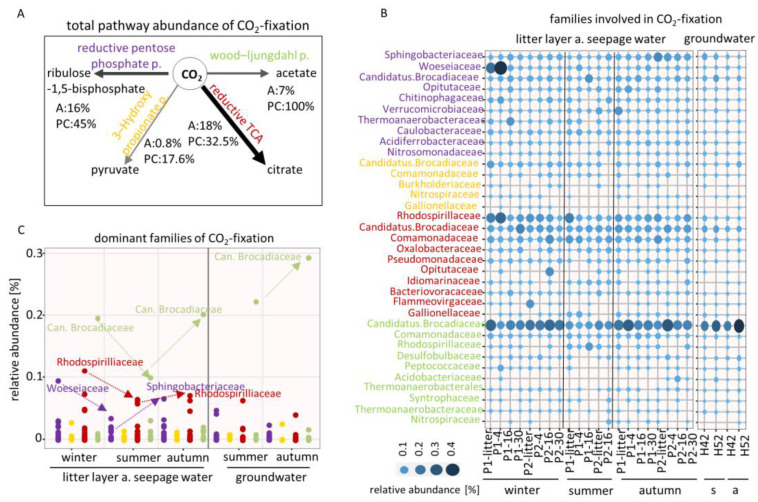
(**A**) Pathway abundance of the CO_2_-fixation across the samples. A = abundance; PC = pathway coverage, calculated by identified number of proteins divided by total proteins per pathway. (**B**) Relative abundances of the families involved in CO_2_-fixation. Colors represent the pathway affiliation; s = summer, a = autumn. P1 and 2 = plot1 and 2. (**C**) Relative abundances of the dominant families involved in CO_2_-fixation in respect of the different seasons.

CO_2_ was mainly fixed by reductive TCA (18%, PC: 32.5%), followed by the reductive pentose phosphate cycle (16%, PC: 45%) and the Wood–Ljungdahl pathway (7%, PC: 100%), while the least common pathway was the 3-hydroxypropionate pathway (0.8%, PC: 17.6%) (Figure 6A). The CO_2_-fixation strategies were diversely distributed over the identified families, while microbes that fix atmospheric carbon were found with higher abundances especially in seepage water (Figure 6B).

CO_2_-fixation by reductive pentose phosphate pathway, a major route of CO_2_ assimilation in most phototrophic bacteria [45,46] dominated by *Woeseiaceae* in early winter (0.1%) with an abundance decrease under drought conditions in summer with fructose-bisphosphate aldolase, class I (K01632), while *Sphingobacteriaceae* dominated in autumn (0.07%) after the rewetting event with glyceraldehyde 3-phosphate dehydrogenase (K00134) (Figure 6C). The reductive tricarboxylic acid (TCA) cycle was dominated by *Rhodospirilliaceae* with an abundance decrease under drought conditions (0.11% to 0.06%) for which we identified isocitrate dehydrogenase (K00031) and aconitate hydratase 2/2-methylisocitrate dehydratase (K01682). We further observed that *Candidatus Brocadiaceae* dominated the Wood–Ljungdahl pathway in seepage water (0.19% and 0.21%) and groundwater (0.29%) under moisture conditions in winter and rewetting conditions in autumn and revealed an abundance decrease under drought conditions. This dominance of *Candidatus Brocadiaceae* is indicated by the abundance of the acetyl-CoA decarbonylase/synthase complex subunit delta and gamma (K00194 and K00197), the anaerobic carbon-monoxide dehydrogenase catalytic and iron sulfur subunit (K00198 and K00196), the acetyl-CoA synthase (K14138), the formate dehydrogenase beta subunit (K15022) and the 5-methyltetrahydrofolate corrinoid/iron sulfur protein methyltransferase (K15023).

## 4. Discussion

### 4.1. Spatial-Temporal Distribution of the Community

A comparison of the microbial community from different seasons and different subsurface sampling sites showed that the microbial community is more influenced by the seasonal transition than by subsoil depth. It is known that autumn litterfall alters nutrient and organic matter in broadleaved forests leading to a seasonally dependent availability of present substrates in the subsurface and, therefore, to an altered community structure [47]. The community remained stable in the first few soil centimeters due to a constant distribution of the nutrient content over the topsoil. In groundwater, we also observed a clear seasonal separation of the microbial community. Seasonal patterns of groundwater were proposed by a microbial community of an oligotrophic alpine groundwater recharge due to the seasonal hydrochemical dynamics reaching the groundwater [48]. The composition of dissolved organic matter (DOM) in the respective wells also showed seasonal shifts, which could reflect changing groundwater connectivity. Microbial-derived DOMs changed during the seasonal transition, reflecting a varying groundwater community on the proteome level. In addition, the groundwater wells are spatially separated predominantly due to differences in their hydrochemical composition since the wells are separated at about 1.4 km from each other [35]. Especially the groundwater well H52 contains higher concentrations of K^+^, Na^+^ and Mg^2+^ compared to well H42, which indicates that hydrochemical conditions impacting the microbial community at different groundwater sites. In a former study, 16S data from the Hainich groundwater were grouped in different community clusters. This also represents spatial effects on the community, which are mainly driven by different specific hydrochemical composition along the transect [49].

### 4.2. Microbial Community Composition is Changed between Seepage Water and Groundwater

*Proteobacteria* (36%) are the most dominant phylum in seepage and groundwater. This is consistent with the results of previous studies where *Proteobacteria* were characterized as the most abundant phylum in subsurface communities [50,51]. *Proteobacteria* are the most diverse microbial phylum comprising phototrophs, autotrophs and heterotrophs; and contain an enormous functional repertoire, which leads to a large functional diversity and thus to their involvement in central ecological processes [52]. However, the seepage water, which flows through the different complex soil layers until it reaches the aquifer, favors various microbes that are dominant in the increasing subsurface depths. We observed that *Microcoleaceae* belonging to the phylum *Cyanobacteria* were overrepresented in seepage water of the topsoil horizon, including predominantly phototrophic microbes. The water in the uppermost soil layer, close to the surface, possibly favors the presence of photosynthetically active bacteria, originating from the rainwater transferred from the organic layer to the upper mineral soil layer [53]. Then, the seepage water percolating deeper into the aquifer favors the strong presence of other bacteria even at far distant sites, due to differences in the hydrochemical composition of soil in increasing subsurface depths. We observed that *Candidatus Brocadiaceae* was more dominant in groundwater, which was also suggested by Starke et al. 2017 [38]. The nitrate-enriched groundwater may indicate the occurrence of nitrate-producing microbes of *Planctomycetes* including *Candidatus Brocadiaceae* [54]. Moreover, this nitrifying family is more abundant in the groundwater (well H52) because this well contains higher NH_4_^+^ concentrations, which are used as an electron donor for nitrate production [35]. The vertical seepage water flow transports transient microbes detached from the surface. Thus, we can only identify a portion of the entire community living in the subsurface environment. A recent study observed that about 45% of the rock-matrix-associated genera were transient and re-dispersed to attached microbes [55]. Thus, in our data, bacterial families including *Gemmatimonadaceae* (phylum: *Gemmatimonadetes*), *Anaerolineaceae* (*Chloroflexi*) and *Chitinophagaceae* (*Cacterioidetes*) are mainly found in nutrient-rich rhizoplane soils or sediments and are underrepresented in our analysis [56,57]. The vertically transferred seepage water and the deeper groundwater are characterized by oligotrophic conditions, which may hamper these underrepresented bacterial families to successfully compete with chemolithoautotrophs, specialized bacteria in nutrient-poor conditions [58]. However, the decreasing evenness until autumn in groundwater community indicates that the dominance of a few specialized microbial families were favored at the seasonal transition from drought to rewetting conditions. In contrast, for topsoil seepage water community, a higher evenness value due to year-round fluctuations, indicates that the seepage hosts fewer-dominant families, which is related to a functional stable microbial community. Such relation of evenness to functional stability was also reported in [59]. Noticeably, we observed that the group *Heterogeneous* showed a mean relative abundance of 19.2% (not shown). This group represents the abundance of protein groups that were not assigned to a unique family because of the protein inference problem [60]. This problem increases with a higher taxonomic resolution (kingdom to species) and increasing community complexity [34]. In addition, a multi-omics approach is a prominent strategy to provide deeper insight into the taxonomic composition of microbial communities by combining metaproteomics with other omics disciplines [61]. 

### 4.3. Seasonal Transition Promotes the Adaption of Microbial Dominances Responsible for Nutrient Cycles

We observed that *nitrogen metabolism* and *carbon fixation pathways* are among the 10% most abundant metabolisms, which includes housekeeping pathways for microbial energy production and thus for their survival and possible dominance within the community. The deep aquifer is a hotspot for microbes involved in the nitrogen cycle since the groundwater represents a nitrate-rich environment [29]. We found nitrogen cycling pathways more abundant in groundwater compared to seepage. The nitrification process, a central pathway of the nitrogen cycle for nitrate production [38,62,63] was dominated by *Nitrosomandaceae* and *Candidatus Brocadiaceae*. Hence, *Nitrosomandaceae* is a common ammonia oxidizing bacteria (AOB) that contains the *amo* and *hao* genes and thereby produces the enzymes for the first step of nitrification to oxidize ammonia to nitrite. There is currently no evidence that *Nitrosomandaceae* is also involved in the second step of nitrification, the oxidation of nitrite to nitrate. Microbes that are suitable for complete ammonia oxidation called *comammox*-bacteria have so far been predominantly found in the family group *Nitrospirae* [64]. Although *Candidatus Brocadiaceae* is a prominent family containing candidates for anammox, it has been shown that it can also be involved in nitrification [38]. The change in dominance of these two families during drought and rewetting conditions, i.e., *Nitrosomandaceae* dominated in summer, while *Candidatus Brocadiaceae* dominated in autumn suggests that these chemolithotrophs adapted to seasonal changed physicochemical conditions in groundwater [65]. The gene *amoA* which is expressed by ammonia-oxidizing bacteria (AOB) was found in the dry months suggesting that microbes that use ammonia as an electron donor including *Candidatus Brocadiaceae*, are more likely to be favored under moist periods [66]. Moreover, *Candidatus Brocadiaceae* dominated the anaerobic ammonium oxidation (anammox) and the denitrification process under rewetting conditions in autumn for the direct removal of nitrate from the subsurface. This is in agreement with the literature where *Candidatus Brocadiaceae* was found as a key family responsible for the anammox process in the subsurface [67]. *Aquiferaceae* was found as the second most-dominant for denitrification and coincides with a former study that revealed the genomic repertoire for denitrification [68]. The CO_2_-fixation, considered as the main strategy for energy recovery of chemolithoautotrophic bacteria, revealed as most abundant in seepage water regarding all four CO_2_-fixation pathways. The near-surface seepage water enables microbes to reach atmospheric CO_2_ for fixation and assimilation in their own metabolism. A recent study found that CO_2_-fixing microbes are also active in groundwater [32], which is consistent with our data since we also identified CO_2_-fixation in both groundwater wells. The metabolic function of the anammox bacteria *Candidatus Brocadiaceae* was also linked to CO_2_-fixation used for carbon assimilation [69], as it dominated the Wood–Ljungdahl pathway in seepage water and groundwater in each season. However, this suggests that *Candidatus Brocadiaceae* is not only responsible for anammox and nitrification especially under rewetting conditions, but also specialized in autotropic CO_2_-fixation during acetogenesis [70]. Thus, the energy production by this potential mixotrophic lifestyle can be adapted depending on the nutrient availability. These two strategies seem to change during the seasonal transition, i.e., in summer *Candidatus Brocadiaceae* may favor CO_2_-fixation to acetate, while in autumn it prefers anammox or nitrification by the transformation of ammonia. Another way to fix CO_2_ is the reductive pentose phosphate pathway for the assimilation of inorganic carbon with the key enzyme ribulose-1,5-bisphosphate carboxylase/oxygenase (RubisCO), the most abundant enzyme worldwide [30]. The dominated presence of *Woeiaceae* for CO_2_-fixation at the beginning of the year is supported by a previous study which confirmed the involvement of *Woeiaceae* in the reductive pentose phosphate pathway by revealing their genomic repertoire [71]. A rewetting event after a drought period may have led to increasing ecological niches within the water saturated topsoil, which have led to a subsequent change in dominance to *Sphingobacteriaceae* in autumn at the end of the year.

## 5. Conclusions

Metaproteomics allows us to characterize the microbial community composition of the Hainich CZE subsurface. Our results indicate that the community composition changes regarding spatial differences of the topsoil seepage water and the deeper and distantly located groundwater sites. We also have found temporal differences regarding seasonal transition on the taxonomy and functionality of the community. We found that changing drought to rewetting periods led to alterations of dominances of a few bacterial families involved in the nitrogen cycle and CO_2_-fixation strategies. Therefore, the seasonal transition prefers the dominant adaption of different bacterial families according to seasonal dynamics. Understanding the functional properties of subsurface microbial communities and their key players involved in central nutrient cycles at the proteome level can help to predict future consequences on the ecosystem functioning.

## Supplementary Materials

The following are available online at https://www.mdpi.com/2076-2607/8/11/1694/s1, Figure S1: Global view on the dissolved organic matter (DOM) in groundwater. Table S1: Overview of lysimeter sampling parameters. Table S2: List of proteins and their quantitation.
