# Supplementary material for: Seasonal Patterns of Dominant Microbes Involved in Central Nutrient Cycles in the Subsurface
Source: Microorganisms. 2020 Oct 30;8(11):1694. doi: 10.3390/microorganisms8111694 (PMC7716230; doi:10.3390/microorganisms8111694)
Supplement: Supplementary file 1 [file microorganisms-08-01694-s001.zip › supplementary_material_figure S1_DOM composition.docx]

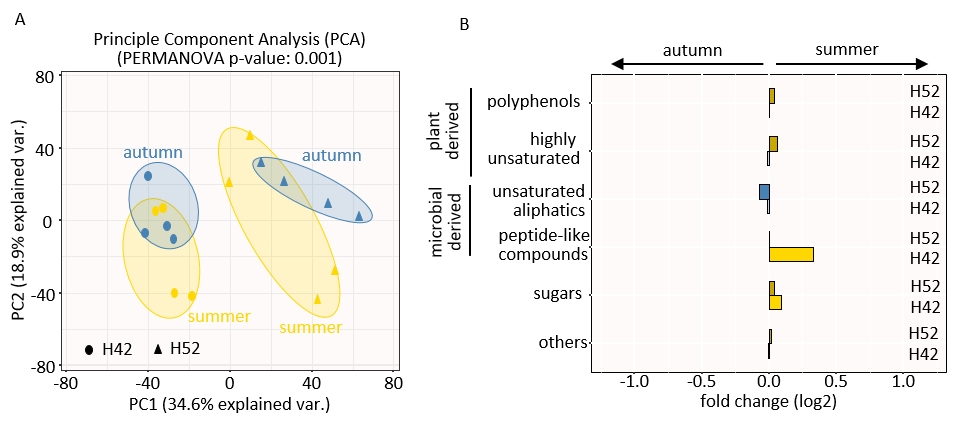


**Figure S1.** (A) Principle component analysis (PCA) of DOM composition of mean abundances from summer and autumn of 2014-2017. (B) Fold change representing classes of DOM compounds which are dominantly found in summer or autumn based on log2 (relative abundance).
