# Supplementary material for: Seasonal Patterns of Dominant Microbes Involved in Central Nutrient Cycles in the Subsurface
Source: Microorganisms. 2020 Oct 30;8(11):1694. doi: 10.3390/microorganisms8111694 (PMC7716230; doi:10.3390/microorganisms8111694)
Supplement: Supplementary file 1 [file microorganisms-08-01694-s001.zip › supplementary_material_table S1_sampling_parameter.docx]

**Table S1:** Overview of lysimeter sampling parameters

| lysimeter | depth (cm) | season | filter volume (ml) | replicate |
| --- | --- | --- | --- | --- |
| P1-litter | 0 | winter | 400 | 1 |
| P1-4 | 4 | winter | 400 | 1 |
| P1-16 | 16 | winter | 400 | 1 |
| P1-30 | 30 | winter | 400 | 1 |
| P2-litter | 0 | winter | 364 | 1 |
| P2-4 | 4 | winter | 150 | 1 |
| P2-16 | 16 | winter | 392 | 1 |
| P2-30 | 30 | winter | 102 | 1 |
| P1-litter | 0 | winter | 400 | 2 |
| P1-4 | 4 | winter | 304 | 2 |
| P1-16 | 16 | winter | 400 | 2 |
| P1-30 | 30 | winter | 156 | 2 |
| P2-litter | 0 | winter | 400 | 2 |
| P2-4 | 4 | winter | 400 | 2 |
| P2-16 | 16 | winter | 400 | 2 |
| P2-30 | 30 | winter | 400 | 2 |
| P1-litter | 0 | summer | 205 | 1 |
| P1-4 | 4 | summer | 120 | 1 |
| P1-16 | 16 | summer | 215 | 1 |
| P2-litter | 0 | summer | 215 | 1 |
| P2-16 | 16 | summer | 215 | 1 |
| P1-litter | 0 | summer | 235 | 2 |
| P1-16 | 16 | summer | 115 | 2 |
| P2-litter | 0 | summer | 96 | 2 |
| P2-16 | 16 | summer | 41 | 2 |
| P1-litter | 0 | autumn | 95 | 1 |
| P1-4 | 4 | autumn | 220 | 1 |
| P1-16 | 16 | autumn | 280 | 1 |
| P1-30 | 30 | autumn | 176 | 1 |
| P2-litter | 0 | autumn | 170 | 1 |
| P2-4 | 4 | autumn | 118 | 1 |
| P2-16 | 16 | autumn | 44 | 1 |
| P2-30 | 30 | autumn | 12.5 | 1 |
| P1-litter | 0 | autumn | 154 | 2 |
| P1-4 | 4 | autumn | 42 | 2 |
| P1-16 | 16 | autumn | 200 | 2 |
| P1-30 | 30 | autumn | 154 | 2 |
| P2-litter | 0 | autumn | 240.5 | 2 |
| P2-4 | 4 | autumn | 129 | 2 |
| P2-16 | 16 | autumn | 139 | 2 |
| P2-30 | 30 | autumn | 162 | 2 |
