# Supplementary figures and images for: Seasonal Patterns of Dominant Microbes Involved in Central Nutrient Cycles in the Subsurface
Source: Microorganisms. 2020 Oct 30;8(11):1694. doi: 10.3390/microorganisms8111694 (PMC7716230; doi:10.3390/microorganisms8111694)

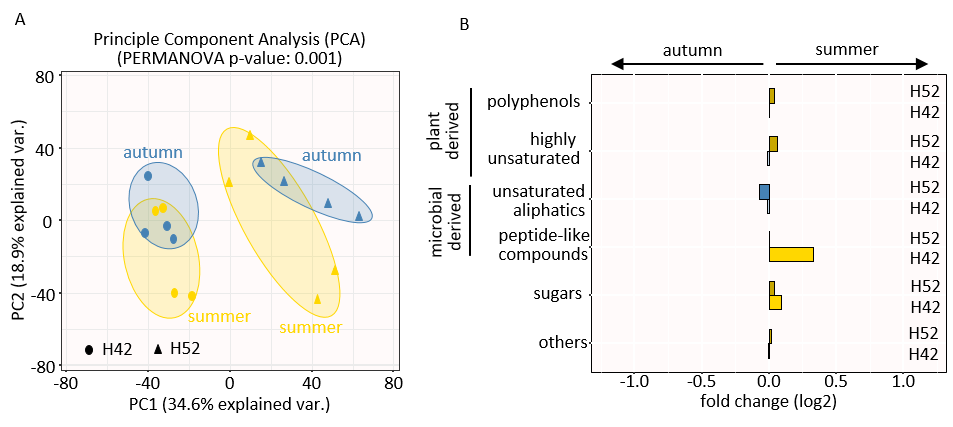

Supplement: Supplementary file 1 [file microorganisms-08-01694-s001.zip › supplementary_material_figure S1_DOM composition_revised.png]
